# Biomechanical Effects of a Novel Pedicle Screw W-Type Rod Fixation for Lumbar Spondylolysis: A Finite Element Analysis

**DOI:** 10.3390/bioengineering10040451

**Published:** 2023-04-07

**Authors:** Jo-Hsi Pan, Chen-Sheng Chen, Chien-Lin Liu, Po-Hsin Chou

**Affiliations:** 1 Institute of Physical Therapy and Assistive Technology, National Yang Ming Chiao Tung University, Taipei 112, Taiwan; 2 Department of Rehabilitation, Cardinal Tien Hospital, New Taipei 231, Taiwan; 3 Department of Orthopedic and Traumatology, Taipei Veterans General Hospital, Taipei 112, Taiwan; 4 School of Medicine, National Yang Ming Chiao Tung University, Taipei 112, Taiwan

**Keywords:** Dynesys stabilization system, finite element models, lumbar spondylolysis, pedicle screw, posterolateral fusion, W-type rod fixation

## Abstract

Lumbar spondylolysis involves anatomical defects of the pars interarticularis, which causes instability during motion. The instability can be addressed through instrumentation with posterolateral fusion (PLF). We developed a novel pedicle screw W-type rod fixation system and evaluated its biomechanical effects in comparison with PLF and Dynesys stabilization for lumbar spondylolysis via finite element (FE) analysis. A validated lumbar spine model was built using ANSYS 14.5 software. Five FE models were established simulating the intact L1–L5 lumbar spine (INT), bilateral pars defect (Bipars), bilateral pars defect with PLF (Bipars_PLF), Dynesys stabilization (Bipars_Dyn), and W-type rod fixation (Bipars_Wtyp). The range of motion (ROM) of the affected segment, the disc stress (DS), and the facet contact force (FCF) of the cranial segment were compared. In the Bipars model, ROM increased in extension and rotation. Compared with the INT model, Bipars_PLF and Bipars_Dyn exhibited remarkably lower ROMs for the affected segment and imposed greater DS and FCF in the cranial segment. Bipars_Wtyp preserved more ROM and generated lower stress at the cranial segment than Bipars_PLF or Bipars_Dyn. The injury model indicates that this novel pedicle screw W-type rod for spondylolysis fixation could return ROM, DS, and FCF to levels similar to preinjury.

## 1. Clinical Significance

In comparison with PLF and Dynesys stabilization, pedicle screw W-type rod fixation preserves more functional ROM and moderates adjacent disc stresses, thereby reducing the risk of adjacent segment disease after surgery.

## 2. Introduction

Spondylolysis is defined as a bone defect of the pars interarticularis [1] that often occurs in the lumbar spine, with a prevalence of 6% in the general population [2]. Lumbar spondylolysis presents clinically as a limited lumbar range of motion (ROM) combined with low back pain [3]. Most patients with lower back pain require sick leave or reduced workloads, leading to economic losses [4,5]. Lumbar spondylolysis is highly prevalent and detrimental; accordingly, efforts should be made to improve treatment methods.

Surgery such as direct pars repair, traditional instrumented fusion, or dynamic fixation is recommended if conservative treatment has failed [6,7,8]. Direct pars repair surgery can reduce pain, speed healing, decrease the risk of degeneration, and enable patients to resume normal activities [8,9]. However, pars repair surgery can only address pain originating from the pars interarticularis [9]. Posterolateral fusion (PLF) is a traditional form of lumbar fusion surgery widely used to treat degenerative spondylolysis [10,11,12]. Clinical investigations report improved clinical [11] and functional [12] outcomes compared with baseline in patients 2 years after undergoing PLF surgery.

Despite these promising results, traditional lumbar fusion surgeries may limit ROM, leading to adjacent segment disease (ASD) [13,14,15]. ASD causes disc degeneration, disc herniation, spondylolisthesis, lumbar instability, arthritis, and stenosis [1]. Accordingly, dynamic lumbar surgery was developed to avoid these complications. The dynamic Dynesys stabilization system (Zimmer Biomet, Warsaw, IN, USA) is a common pedicle screw-based dynamic instrumentation device that uses polyethylene–terephthalate cords with polycarbonate–urethane spacers to connect the screws for fixation [16]. The polymer has high elasticity, which preserves some ROM and reduces the risk of ASD [16]. Schnake et al. demonstrated that Dynesys implantation can be achieved without bone grafting, thereby eliminating the risk of donor site morbidity [17]. These studies further demonstrated the clinical benefits of dynamic lumbar stabilization surgery.

Wang et al., using finite element (FE) analysis, found that bilateral pars fractures increased vertebral body displacement, disc stress, endplate stress, and the posterior ligament force of the affected segment under extension and torsion, increasing the risk of early disc degeneration [18]. Han et al. created FE models of an intact lumbar spine (INT) and a spine with PLF and reported that PLF can stabilize the lumbar spine [19]. Demir et al. [20], in a similar study using a model of the Dynesys system, demonstrated that the Dynesys system can provide stability. Although these two studies demonstrated that PLF and the Dynesys system can stabilize the lumbar spine, no studies have examined the suitability of PLF and Dynesys for the treatment of lumbar spondylolysis. Accordingly, the present study investigated whether PLF and Dynesys stabilization are effective interventions for pars fractures.

**Figure 1:**
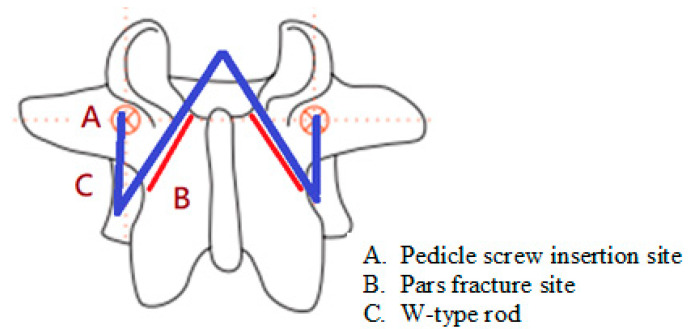
Bilateral pars fractures with pedicle screw W-type rod fixation. Note: red line indicates the fracture site; and blue line indicates the shape of the W-type rod.

We introduced a novel pedicle screw W-type rod fixation technique, which is performed as follows. First, titanium screws are inserted into the pedicles of the affected segment. Next, the screws are connected to a W-type rod with the hypotenuse parallel to the fracture site (Figure 1). In traditional treatment, PLF surgery changes the loading environment because the rod connects two vertebral bodies and scarifies its motion segment. As result, PLF surgery is found in early adjacent disc degeneration. The Dynesys system also has similar questions despite there being a rigid rod instead of soft space. The preserved movement of the affected motion segment is limited, so the clinical study addressed an iatrogenic effect on the adjacent disc. The novelty of the pedicle screw W-rod is that it can undergo local fixation around the pars fracture other than having a large fixation such as Dynesys system or PLF surgery. In fact, the lumbar spine with a pars fracture causes the spinal posterior element to break into three pieces, consisting of one posterior lamina and two superior facet joints. The W-rod was designed to connect the three pieces into a complete spinal posterior element. We hypothesized that the hypotenuse of the W-rod would compensate for the reduced ability of the fractured pars to transmit force and that, because the screws were inserted only in the affected segment, this method has the potential to preserve more ROM than traditional PLF surgery or the dynamic Dynesys system.

To verify our hypothesis, we used FE analysis to determine the biomechanical effects on lumbar spondylolysis of PLF, the Dynesys system, and pedicle screw W-type rod fixation. The objective of the study was to examine the performance of pedicle screw W-type rod fixation in terms of ROM, disc stress, and facet joint contact force among three FE models.

## 3. Material and Methods

We performed FE analyses to investigate the biomechanical changes in lumbar spondylolysis after intervention with PLF, the Dynesys system, and pedicle screw W-type rod fixation. We constructed five FE spine models consisting of five vertebral bodies and four discs representing L1–L5. FE analyses were conducted using the commercial version of ANSYS 14.5 (ANSYS, Canonsburg, PA, USA) to model the lumbar spine. Model descriptions, loading conditions, and boundary conditions are described in the following sections.

### 3.1. Intact Lumbar Spine

**Figure 2:**
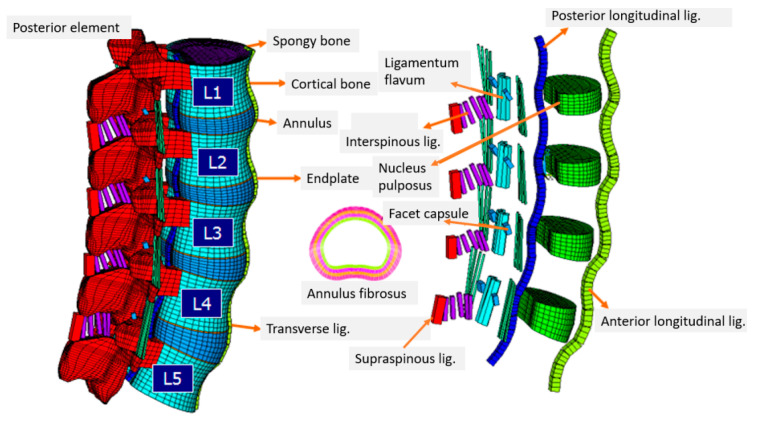
Finite element model of the intact lumbar spine.

This study used a validated FE spinal model that includes the vertebrae, intervertebral discs, endplates, posterior bony elements, and ligaments (anterior longitudinal, posterior longitudinal, ligament flavum, supraspinous, interspinous, transverse, and capsular) [21]. Most material properties of the model were assumed to be homogeneous and isotropic. The elastic modulus of the ground substance in spinal disc was simulated using a nonlinear hyperelastic, two-parameter (C10, C01) Mooney–Rivlin solid model. C10 and C01 represent the constants used with the FE software. These constants characterized the deviatoric deformation according to the Mooney–Rivlin solid model for material constants C1 and C2, respectively [21,22]. We used two-node link elements to simulate annulus fibers and ligaments that resisted tension only. The contact characteristics of the facet articulation were simulated by three-dimensional contact elements. We assumed the contact condition was initiated when the normal gap distance was less than 1 mm and the friction coefficient was 0.1. The remaining portions of the spine were simulated by eight-node solid elements, including vertebrae, disc nucleus, endplates, and the posterior bony element (Figure 2) [22].

A previous study [21] performed a convergence test using three mesh densities (finest model: 112,174 elements/94,162 nodes; normal model: 27,244 elements/30,630 nodes; coarse model: 4750 elements/4960 nodes) and tested for change in ROM. The researchers ultimately selected the finest mesh density because the change between it with the normal model was within 1.03% in flexion (<0.2°), 4.39% in extension (<0.5°), 0.01% in torsion (<0.2°), and 0.001% in lateral bending (<0.1°) [21]. This validated model was selected as the FE model for the present study.

ROM and facet joint contact force (FCF) were chosen for model validation. First, different moments of 3.75, 7.5, and 10 Nm were applied to the INT model, and ROMs for the five validated levels were determined [23]. ROMs achieved good agreement under most of the loading. Under a 10 Nm moment and 150 N preload, the model exhibited some stiffness in comparison to in vitro tests. Next, we compared the FCF of five levels in rotation with the results of Chen et al. [24] and Shirazi-Adl et al. [25] and found a similar trend. Therefore, the INT model could be used for further analysis.

### 3.2. Lumbar Spine with Bilateral Pars Fractures

**Figure 3:**
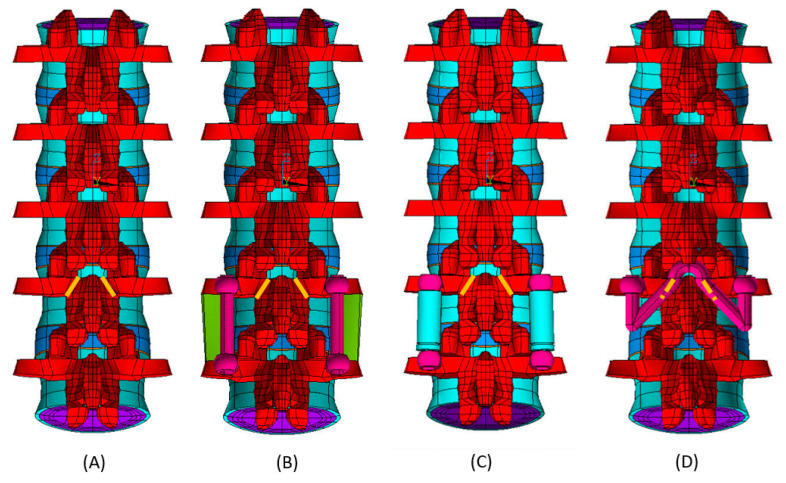
(**A**) Bipars. (**B**) Bipars_PLF. (**C**) Bipars_Dyn. (**D**) Bipars_Wtyp. Note: yellow line indicates the fracture site.

We constructed 0.1 mm gaps at each side of the L4 pars interarticularis in the INT model to mimic spondylolysis and named the new model “Bipars.” According to Lee’s study [26], the direction of the defects was oblique through the isthmus (Figure 3A). The Bipars model comprised 116,831 elements and 95,585 nodes.

### 3.3. Bilateral Pars Fractures with Posterolateral Fusion

The Bipars_PLF model simulated a lumbar spine with spondylolysis, spinal instrumentation, and a bone graft placed between the transverse processes of adjacent vertebrae (Figure 3B). The study modeled bilateral pars fractures at the L4 and fused L4–L5 motion segments. We simulated four titanium alloy pedicle screws (diameter (Φ) = 6 mm, length (L) = 44 mm), and two titanium alloy rods (Φ = 5.4 mm, L = 40 mm) to connect the screws and applied them to the Bipars model; this model was named “Bipars_PLF.” The Bipars_PLF model comprised 276,738 elements and 123,577 nodes. This model assumed that patients would have obtained solid fusion, and consequently, the stress distribution in the model allowed the bone graft to transmit compression and tension force.

### 3.4. Bilateral Pars Fractures with Dynesys System

The Dynesys model simulated a lumbar spine with spondylolysis and spinal instrumentation. The study modeled bilateral pars fractures at the L4 and fused L4–L5 motion segments. We constructed four titanium alloy pedicle screws (Φ = 6 mm, L = 44 mm), two polycarbonate urethane (PCU) spacers (outer Φ = 12 mm, inner Φ = 9 mm, L = 30 mm), and two polyethylene terephthalate (PET) cords (outer Φ = 7 mm, inner Φ = 6 mm, L = 10 mm) and applied them to a fused L4–L5 motion segment in the Bipars model. Subsequently, we applied 300 N of elasticity pretension on the cords; this model was named “Bipars_Dyn.”. The material parameters of the model (Figure 3C) are displayed in Table 1. The Bipars_Dyn model included 158,540 elements and 101,791 nodes.

### 3.5. Bilateral Pars Fractures with Pedicle Screw W-Type Rod Fixation

**Figure 4:**
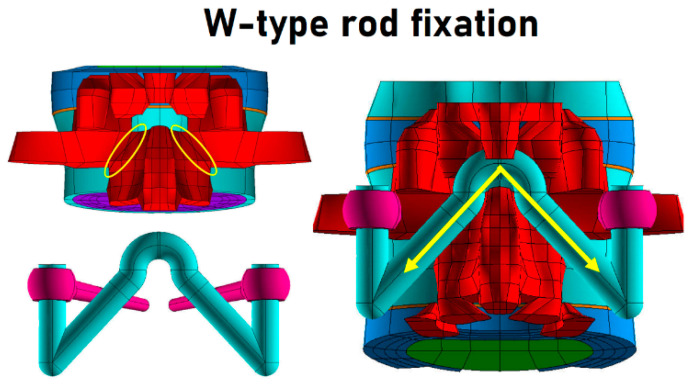
Construct of the W-type fixation. The pars fracture (yellow ellipse) induces spinal instability. The W-type rod (yellow arrow) creates a new loading pathway.

The novel technique required a rod that could connect to pedicle screws and lay parallel to the fissure site to help the fractured pars transmit force and improve its stability. The rod meeting these conditions was a W-type rod (Figure 3D and Figure 4). Apart from stability, the other advantage of W-type rod fixation is that the rod can be removed to facilitate fusion surgeries when spondylolysis eventually progresses to spondylolisthesis.

The pedicle screw W-type rod fixation model included a lumbar spine with bilateral pars fractures and spinal instrumentation. We set up two titanium alloy pedicle screws (Φ = 6 mm, L = 44 mm) at the L4 vertebrae and connected them to a titanium alloy rod (Φ = 5.5 mm) folded into a “W” shape in the Bipars model and named the new model “Bipars_Wtyp.” The hypotenuse of the W-type rod was parallel to the fracture sites. The Bipars_Wtyp model included 261,188 elements and 121,664 nodes.

### 3.6. Boundary and Loading Condition

In the present study, we completely constrained the inferior surfaces of the L5 vertebrae. The loading condition was as follows:

1. A 150 N normal axial load was applied to the superior surface of the L1 vertebrae.
2. Bending moment was applied to the superior surface of the L1 vertebrae using the following parameters: flexion 19.9°, extension 12.3°, lateral bending 22.5°, and rotation 10.9°. The ROM comprised the maximum ranges of all FE models.

### 3.7. Biomechanical Evaluation

Bilateral pars fractures increase lumbar ROM at the affected level, which is why PLF, Dynesys, and W-type rod fixation were developed to solve the problem. However, fusion surgeries may over-limit the ROM. Therefore, the present study analyzed the ROM of the affected segment.

Lumbar fusion surgeries may cause ASD; accordingly, the W-type rod fixation technique was developed to preserve spinal motion and prevent early degeneration of the adjacent discs. Therefore, we needed to pay attention to the intervertebral disc stress and FCF at the adjacent segment.

## 4. Results

Compared to the INT model, the Bipars model was remarkably unstable in extension and rotation, exhibiting ROM increases of 43% and 26%, respectively. In flexion and lateral bending, the ROM differences in the Bipars and the INT models were within 4% of each other.

### 4.1. Flexion

**Figure 5:**
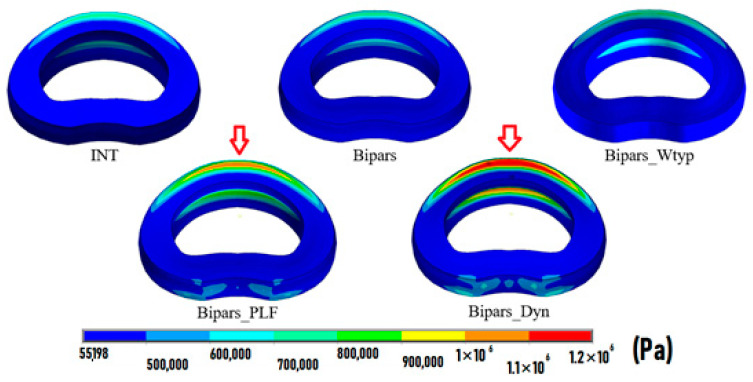
Adjacent disc stress (L3–L4) in flexion. Note: Red arrows indicate points of high stress.

The ROMs for flexion are depicted in Table 2. The Bipars_PLF and Bipars_Dyn models were remarkably over-limit, with decreases of 65% and 77%, respectively, compared with the INT model. The Bipars_Wtyp model was 8% lower than the INT model. The stress in the disc annulus at the cranial segment increased by 40% and 51% in the Bipars_PLF and Bipars_Dyn models, respectively. High stress was especially concentrated at the anterior disc edge (Figure 5). Additionally, the traditional treatment such as Bipars_PLF and Bipars_Dyn also received more stress on the posterior region of the disc than the Bipars_Wtyp. It meant that the Bipars_PLF and Bipars_Dyn did change the stress flow of the lumbar spine. In the Bipars_Wtype model, disc stress was only 4% higher than in the INT model. The facet joints would open during flexion, and therefore, the FCF in all models was zero.

### 4.2. Extension

The ROMs for extension are depicted in Table 3. The Bipars_PLF and Bipars_Dyn models were remarkably over-limit, with decreases of 73% and 38%, respectively, compared with the INT model. The Bipars_Wtype model could provide partial stability, with a ROM 13% lower than in the Bipars model.

**Figure 6:**
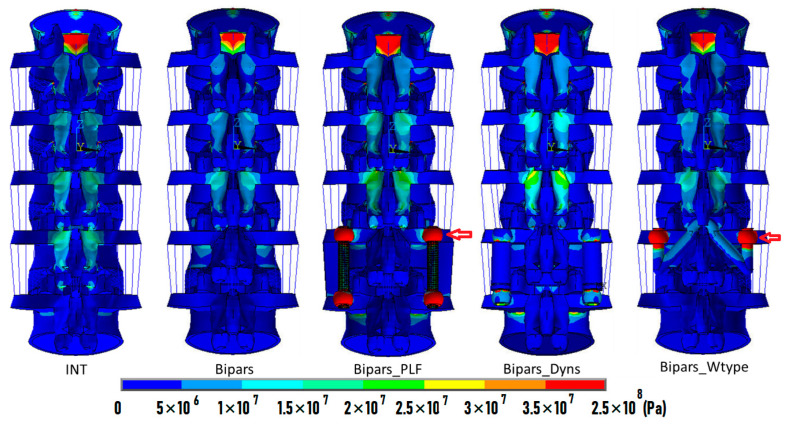
Stress distribution in all FE models in extension. High stress at screw heads was observed in the Bipars_PLF and Bipars_Wtyp models as indicated by the red arrows.

Focusing on the spinal instrumentation, we found that the Bipars_PLF model absorbed the most stress, and the W-type instrumentation helped the fractured pars transmit force (Figure 6). Conversely, the Bipars_Dyn model absorbed the least stress because of the soft PCU spacer. It meant that a metal W-type rod guided stress from pedicle to pedicle instead of fracture pars on the spinal lamina.

**Figure 7:**
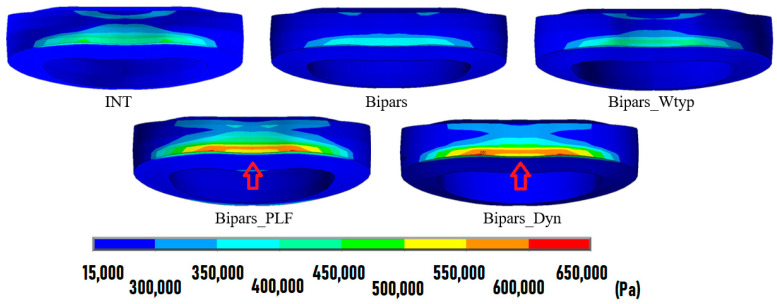
Adjacent disc stress (L3–L4) in extension. Note: Red arrows indicate points of high stress.

In the Bipars_PLF and Bipars_Dyn models, the disc stress of the cranial segments increased by 29% and 19%, respectively, compared with the INT model. In the Bipars_Wtyp model, disc stress was 8% lower than in the INT model. Apparently, the high stress acted on the posterior region of the adjacent disc in the Bipars_Dyn and Bipars_Wtyp model, as shown in Figure 7. Minor changes were observed in the INT and Bipars_Wtyp model. It could be explained why early adjacent disc degeneration was observed in the B_PLF and Bipars_Dyn treatment. The adjacent segment FCFs were increased in the Bipars_PLF and Bipars_Dyn models by 32% and 19%, respectively; this value decreased in the Bipars_Wtyp model.

### 4.3. Lateral Bending

The ROMs for lateral bending are depicted in Table 4. The Bipars_PLF and Bipars_Dyn models were remarkably low, with decreases of 74% and 51%, respectively, compared with the INT model. The Bipars_Wtyp ROM was 13% lower than that of the INT model. Cranial segment disc stress increased in the Bipars_PLF and Bipars_Dyn models by 21% and 13%, respectively, but the greatest FCF occurred in the Bipars_PLF model.

### 4.4. Rotation

The ROMs for rotation are depicted in Table 5. The Bipars_PLF model’s ROM was remarkably over-limit, with a decrease of 443% compared with the INT model. The Bipars_Dyn and Bipars_Wtyp models provided partial stability, with ROMs 16% and 11% lower than the Bipars model, respectively. In the Bipars_PLF model, cranial segment disc stress and ipsilateral FCF were 26% and 39% higher than in the INT model, respectively.

## 5. Discussion

Lumbar spondylolysis may result in limited ROM, lower extremity pain, lower back pain, and economic loss [3,4,5]. It also causes biomechanical impairments. In 2010, Fan et al. used calf specimens to analyze the changes in ROM after bilateral pars fractures and discovered that ROM increased substantially to 132.1% in flexion–extension and to 148.8% in axial rotation, but no substantial change occurred in lateral bending [27]. In Wang’s FE study [19], the Bipars model was remarkably unstable in extension and rotation, which increased by 206% and 260%, respectively, compared with the INT model. Our study corroborated these findings, revealing instabilities in the extension and rotation of the lumbar spine. However, a unique finding of our study is that the intact articular ligament between the disc and thoracic construct can counter movement during lateral flexion [27], rendering the ROM changes insignificant.

Studies have demonstrated that traditional lumbar fusion surgeries can limit the ROM of the affected segment, leading to ASD [13,14,15,28]. The present study revealed that the change in ROM is the same in the lumbar spondylolysis group. We also observed that the PLF-treated lumbar spine with bilateral pars fractures imposed greater stress on the discs and facet joints of adjacent segments. This stress could be the mechanism responsible for the accelerated disc degeneration and the hypertrophic degenerative arthritis of the facet joint that occurs in patients with PLF [29]. Although PLF improves stability in spines affected by pars fracture, it also induces an iatrogenic effect at the motion segment adjacent to the fusion site.

Kim et al. [30] and Schaeren et al. [31] concluded that the Dynesys stabilization system causes stress at the cranial segment and cannot prevent ASD. Our study corroborated these results. We found that the spondylolysis lumbar spine with Dynesys stabilization, when compared with an intact lumbar spine, imposed greater stress on the discs and facet joints of the cranial segment during flexion, extension, and lateral bending. However, this effect was not observed during rotation, possibly because cords and spacers cannot resist torsion; therefore, the ROM of the affected segment is preserved, and the cranial segment does not need to compensate. Additionally, the Dynesys system was designed to reduce stiffness and preserve greater ROM at the fusion segment than traditional lumbar fusion surgery [32]. Our study demonstrated that the ROM of the Dynesys model was greater than that of the PLF model, except in flexion. This is probably due to the pretension (300 N) of the cord increasing the stiffness of the Dynesys system during flexion movements. This study aimed to determine whether the Dynesys system could be applied to treating pars fractures, and we concluded that Dynesys could provide enough stability and preserve more rotation ROM compared with traditional fusion surgery.

**Figure 8:**
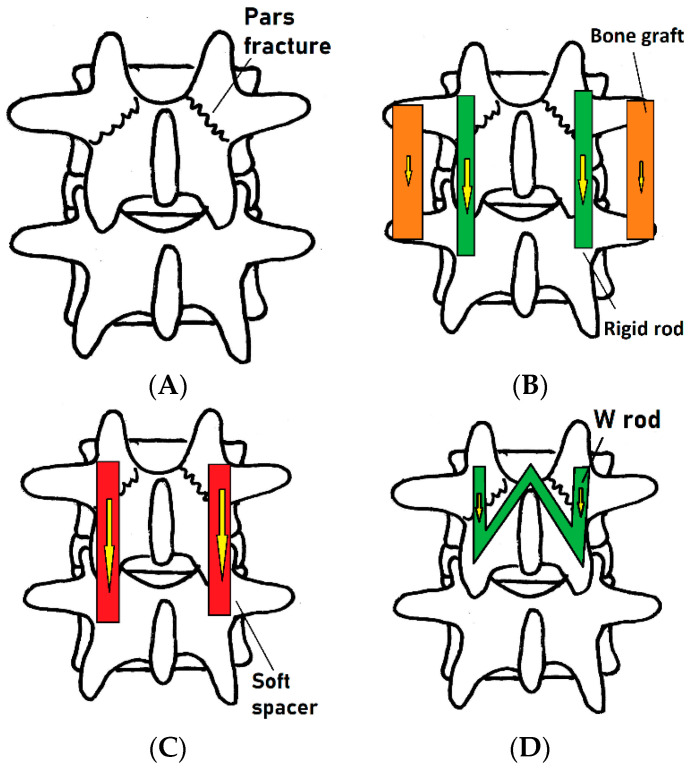
Force flow (arrow indicated) of spinal motion segment in four FE models: (**A**) Bipars, (**B**) Bipars_PLF, (**C**) Bipars_Dyn, (**D**) Bipars_Wtyp.

We hypothesized that pedicle screw W-type rod fixation could preserve more ROM than PLF and Dynesys stabilization for lumbar spondylolysis, and this hypothesis was verified in our simulations, probably because the novel technique does not require fusion of the affected segment to the adjacent level. The ROM of the Bipars_Wtyp model resembled the INT model in flexion, lateral bending, and rotation, meaning that this technique can increase stability in lumbar spines affected by bilateral pars fractures. Moreover, the ROMs of the Bipars_Wtyp model were approximately 19% lower than those of the Bipars model, indicating that the novel pedicle screw W-type rod fixation technique can partially stabilize lumbar spines affected by pars fracture. In traditional treatment, the PLF and Dynesys stabilizations are able to strengthen spinal stability, but they also scarify spinal movement on the affected segment and cause early adjacent disc degeneration. In fact, the pars fracture caused breakage of the loading pathway on the lamina of the lumbar spine. In the study, we only created a new loading pathway such as a metal W-type implant to transmit force on the lamina. The W-type rod fixation fixes pars fracture without affecting a motion segment. However, the Dynesys and PLF fixations span two vertebral bodies with one disc and scarify movement of one spinal motion segment, as shown in Figure 8. However, the W-rod fixation indirectly repaired the pars fracture and, thus, preserved its normal movement in one motion segment. Additionally, a previous study [33] also conducted a biomechanical comparison of different devices consisting of a pedicle-screw cable construct, pedicle screw-rod-hook construct, U-type interlaminar rod construct, and wiring technique in the treatment of spondylolysis. Their study reported that a U-type achieved better spinal stability in the location of the pars fracture. In this study, the W-type rod fixation is similar to the U-type fixation, so it can obtain a comparable result.

The disc and FCF of the cranial segment of the Bipars_Wtyp model were remarkably lower than those of the Bipars_PLF and Bipars_Dyn models, which suggests that this technique offers decreased risks of ASD in comparison with fusion surgeries. This may be because the pedicle screw W-type rod fixation technique preserves more ROM and the hypotenuse of the rod can compensate for the fractured pars to transmit force. The W-type rod can also be removed to facilitate fusion surgeries at a later date should disc degeneration occur.

In an in vitro study, it is difficult to obtain a cadaveric specimen and the individual difference that exist in a clinical experiment. Additionally, the stress flow of the lumbar spine, while giving to external moment, is difficult to be visualized in the in vitro study [34,35]. The FE technique was able to design a new orthopedic device and analyze its biomechanical features [36,37,38,39]. Therefore, FE simulation was chosen in the study. However, this FE study has several limitations. First, for simplification of the spinal implant model, the viscoelastic behavior of the PET and PCU materials in the Dynesys system was not considered. Second, the ROMs of the FE models analyzed in this study were below the physiological range because of limitations in the FE model due to mesh convergence. Third, our models did not include muscle tissue, and therefore, the biomechanical effects of the study were similar to those of the in vitro study. Fourth, the lumbar spine was analyzed in von Mises stress. The results were little different to applications of different failure criteria or nonlinear materials [40,41]. Although this study demonstrated good performance of the pedicle screw W-type rod fixation, we still must confirm these results in clinical trials.

## 6. Conclusions

This study demonstrated that bilateral pars fractures can decrease the stability of the lumbar spine in extension and rotation. Pedicle screw W-type rod fixation could provide stability in lumbar spines affected by bilateral pars fractures. This novel technique also preserves greater ROM and reduces stress on discs and facet joints at cranial segments in comparison with PLF and Dynesys stabilization systems. These structural changes may possibly result in the preservation of more functional ROM and a reduction in the risk of ASD in patients with lumbar spondylolysis. Although the study obtained the expected results, an in vitro experiment and clinical evaluation for the W-type rod fixation should be conducted in a future study.

## Figures and Tables

**Table 1:** Material parameters of the Dynesys system.

Material	Element Type	Young’s Modulus (MPa)	Poisson’s Ratio
Titanium alloy screw	8-node SOLID185	110,000	0.28
PCU spacer	8-node SOLID185	68.4	0.4
PET cord	2-node LINK10	1500	0.4

**Table 2:** ROMs, adjacent disc stress, and adjacent FCF in flexion.

	INT	Bipars	Bipars_PLF	Bipars_Dyn	Bipars_Wtype
Loading moment (Nm)	9.6	9.6	12.0	12.3	9.9
L4-L5 ROM (degree)	5.98 (0%)	6.17 (+3%)	2.10 (−65%)	1.37 (−77%)	5.48 (−8%)
L3-L4 adjacent disc stress (KPa)	780 (0%)	772 (−2%)	1100 (+40%)	1190 (+51%)	819 (+4%)
L3-L4 adjacent FCF (N)	0	0	0	0	0

Note: percentages indicate the values of all the models normalized by the corresponding values of the INT model ([Bipars_model − INT_model]/[INT_model]) × 100%.

**Table 3:** ROMs, adjacent disc stress, and adjacent FCF in extension.

	INT	Bipars	Bipars_PLF	Bipars_Dyn	Bipars_Wtyp
Loading moment (Nm)	9.0	7.5	11.7	10.8	8.4
L4-L5 ROM (degree)	3.05 (0%)	4.36 (+43%)	0.81 (−73%)	1.90 (−38%)	3.78 (+24%)
L3-L4 adjacent disc stress (KPa)	473 (0%)	401 (−15%)	612 (+29%)	562 (+19%)	437 (−8%)
L3-L4 adjacent FCF (N)	81 (0%)	64 (−21%)	107 (+32%)	96 (+19%)	72 (−11%)

Note: percentages indicate the values of all the models normalized by the corresponding values of the INT model ([Bipars_model − INT_model]/[INT_ model]) × 100%.

**Table 4:** ROMs, adjacent disc stress, and adjacent FCF in lateral bending.

	INT	Bipars	Bipars_PLF	Bipars_Dyn	Bipars_Wtyp
Loading moment (Nm)	11.7	11.7	14.1	13.2	12.0
L4-L5 ROM (degree)	4.94 (0%)	5.12 (+4%)	1.27 (−74%)	2.42 (−51%)	4.28 (−13%)
L3-L4 adjacent disc stress (KPa)	997 (0%)	991 (−1%)	1210 (+21%)	1130 (+13%)	1030 (+3%)
Left/Right L3-L4 adjacent FCF (N)	12/1	10/0	38/14	22/7	17/4

Note. percentages indicate the values of all the models normalized by the corresponding values of the INT model ([Bipars_model − INT_model]/[INT_model]) × 100%.

**Table 5:** ROMs, adjacent disc stress, and adjacent FCF in rotation.

	INT	Bipars	Bipars_PLF	Bipars_Dyn	Bipars_Wtyp
Loading moment (Nm)	9.9	7.8	13.5	9.0	9.0
L4-L5 ROM (degree)	3.76 (0%)	4.73 (+26%)	2.13 (−43%)	3.96 (+5%)	4.20 (+11%)
L3-L4 adjacent disc stress (KPa)	387 (0%)	338 (−13%)	489 (+26%)	384 (−1%)	374 (−3%)
Left/Right L3-L4 adjacent FCF (N)	0/124	0/90	0/172	0/110	0/113

Note: percentages indicate the values of all the models normalized by the corresponding values of the INT model ([Bipars_model − INT_model]/[INT_model]) × 100%.

## Data Availability

The data presented in this study are available on request from the corresponding author.
